# Combined metabolomics and tandem machine-learning models for wound age estimation: a novel analytical strategy

**DOI:** 10.1093/fsr/owad007

**Published:** 2023-04-25

**Authors:** Jie Cao, Guoshuai An, Jian Li, Liangliang Wang, Kang Ren, Qiuxiang Du, Keming Yun, Yingyuan Wang, Junhong Sun

**Affiliations:** School of Forensic Medicine, Shanxi Medical University, Jinzhong, China; School of Forensic Medicine, Shanxi Medical University, Jinzhong, China; School of Forensic Medicine, Shanxi Medical University, Jinzhong, China; School of Forensic Medicine, Shanxi Medical University, Jinzhong, China; School of Forensic Medicine, Shanxi Medical University, Jinzhong, China; School of Forensic Medicine, Shanxi Medical University, Jinzhong, China; School of Forensic Medicine, Shanxi Medical University, Jinzhong, China; School of Forensic Medicine, Shanxi Medical University, Jinzhong, China; School of Forensic Medicine, Shanxi Medical University, Jinzhong, China

**Keywords:** forensic sciences, forensic pathology, wound age estimation, metabolomics, tandem machine learning, multilayer perceptron

## Abstract

Wound age estimation is one of the most challenging and indispensable issues for forensic pathologists. Although many methods based on physical findings and biochemical tests can be used to estimate wound age, an objective and reliable method for inferring the time interval after injury remains difficult. In the present study, endogenous metabolites of contused skeletal muscle were investigated to estimate the time interval after injury. Animal model of skeletal muscle injury was established using Sprague–Dawley rat, and the contused muscles were sampled at 4, 8, 12, 16, 20, 24, 28, 32, 36, 40, 44, and 48 h postcontusion (*n* = 9). Then, the samples were analysed using ultraperformance liquid chromatography coupled with high-resolution mass spectrometry. A total of 43 differential metabolites in contused muscle were determined by metabolomics method. They were applied to construct a two-level tandem prediction model for wound age estimation based on multilayer perceptron algorithm. As a result, all muscle samples were eventually divided into the following subgroups: 4, 8, 12, 16–20, 24–32, 36–40, and 44–48 h. The tandem model exhibited a robust performance and achieved a prediction accuracy of 92.6%, which was much higher than that of the single model. In summary, the multilayer perceptron–multilayer perceptron tandem machine-learning model based on metabolomics data can be used as a novel strategy for wound age estimation in future forensic casework.

**Key Points:**

- The changes of metabolite profile were correlated with the time interval after injury in contused skeletal muscle.
- A panel of 43 endogenous metabolites screened by ultraperformance liquid chromatography coupled with high-resolution mass spectrometry could distinguish the wound ages.
- The multilayer perceptron (MLP) algorithm exhibited a robust performance in wound age estimation using metabolites.
- The combination of matabolomics and MLP–MLP tandem model could improve the accuracy of inferring the time interval after injury.

## Introduction

In forensic practice, suspect identification has always been a challenge in criminal investigations because of inadequate evidence due to the suspect escaping, cleaning of the scene, or a crime involving multiple people [1, 2]. Violent crimes often occur with physical wounds, and the wound age can indicate the time of the harmful behaviour from the perpetrator in the actual situation. Thus, connecting the time point of physical injury to that of harmful behaviour is considered to be important for suspect identification. Notably, wound age estimation is a crucial step in forensic practice for suspect identification.

In preliminary studies of wound age estimation, only the signs on the body were used by forensic specialists to infer the time interval after injury. Due to the continuous development of detection techniques and methods, increasing evidence has shown that a variety of biological substances such as mRNA, micro-RNA, and proteins can be candidate markers for determining wound age [3–6]. However, no marker has been proven to be efficient and reliable to date.

In recent years, based on the theory that biological processes after tissue injury are comprehensive and complicated, a consensus was made that multiple biomarkers should be applied to estimate wound age [1, 7–11]. Omics technology has provided an approach to study these biological processes from a holistic view. Kondo [12] reviewed several markers for determining skin wound age in forensic medicine and predicted that proteins would be useful for establishing wound vitality or age.

The analysis of low-molecular-weight metabolites, also known as metabolomics, is a systematic study of the metabolic changes in all endogenous small molecules in response to stimuli [13]. Metabolomics may reflect function more directly than the analyses of proteins or genes, which have been successfully applied to several areas of medical research. Current advances in technology allow for the simultaneous characterization of thousands of metabolites. Molecular changes reflecting metabolic changes in mice of different ages and contused human skeletal muscle have been measured in the previous studies, thereby providing the basis for future detailed metabolomics investigations of injured muscle [14, 15].

With the significant growth in data volume, the difficulty of data explosion originated from metabolomics should also be concerned. Fortunately, due to advances in machine learning, building an intelligent model of the distribution of class labels in terms of predictor features is possible. The resulting classifier is then used to assign class labels to test instances for which predictor feature values are known but class label values are unknown. Accordingly, in forensic medicine field, machine-learning algorithms have also been applied to determine the cause of death, infer skeletal age, and estimate the postmortem interval with vitreous humour, drowning fluid, limb long bone, and other biological specimens [16–21]. Despite numerous advances in methodology in the past years, forensic medicine research scientists continue to rely heavily on parametric models (e.g. logistic regression) for prediction. Often, only a single model is specified to generate predictions and, in some multiclassification tasks, the performance of a single model is always unsatisfactory. As we know, the combination of multiple machine-learning models into ensembles to decrease the forecast errors in the forensic medicine field has not been reported in the previous studies.

In the present study, ultraperformance liquid chromatography coupled with high-resolution mass spectrometry (UPLC–HRMS) was used to investigate metabolite changes in contused muscles over time, and a tandem machine-learning algorithm was applied to narrow the prediction time window after skeletal muscle contusion based on the levels of different metabolites.

## Materials and methods

### Animal experiments and sample preparation

All procedures performed conformed to the Guide for the Care and Use of Laboratory Animals (https://nap.nationalacademies.org/catalog/12910/guide-for-the-care-and-use-of-laboratory-animals-eighth) and were approved by the Institutional Animal Care and Use Committee of Shanxi Medical University, China. A total of 117 male Sprague–Dawley rats (7–8 weeks old; weight, 200–250 g) were purchased from the Animal Center of Shanxi Medical University. This study was also approved by the Animal Ethics Committee of Shanxi Medical University (2016LL151). All laboratory animals were housed in cages and fed rat chow and water under a 12-h light/12-h dark cycle and relative humidity of 40%–60% at 22°C–24°C. Then, the 117 rats were randomly divided into a control group and 4-, 8-, 12-, 16-, 20-, 24-, 28-, 32-, 36-, 40-, 44-, and 48-h contusion groups for the experiments (*n* = 9).

The animal model of skeletal muscle contusion has been described previously [22]. Briefly, the rats were anesthetized with pentobarbital sodium and then the hair on their right posterior limbs was removed using a depilatory agent. The rats were placed on a test stand in the supine position. Subsequently, a 100-g counterpoise was dropped from a height of 200 cm through a clear lucite guide tube onto the quadriceps femoris muscle of the right posterior limb. The rats that received the injury were transferred to another clean cage with food and water and were sacrificed at 12 contused time point (4, 8, 12, 16, 20, 24, 28, 32, 36, 40, 44, and 48 h) postcontusion with a lethal dose of pentobarbital sodium (350 mg/kg body weight, intraperitoneal injection). A 200-mg skeletal muscle specimen was dissected from the wound site. The specimens in the control group were harvested from the same site after anesthetization with an overdose of pentobarbital sodium. All muscle samples were frozen immediately in liquid nitrogen and were stored at −80°C until metabolomics analysis.

Finally, another 13 rats representing various wound time points were selected as the test dataset to assess the performance of the final tandem prediction model. The muscle specimen preparation and metabolomics analytical procedures were the same as for the 117 samples mentioned above.

### Metabolite extraction

Muscle samples were prepared by first thawing the specimens at 4°C before extracting 200-mg muscle samples. Each sample was homogenized in an 800-μL aliquot of acetonitrile using the ball-milling method, then centrifuged at 12 000 rpm for 20 min at 4°C. Next, 600 μL of supernatant were transferred to and dried in a refrigerated centrifugal drier. Finally, the dried residue was redissolved with 200 μL of acetonitrile/water (4:1), and the supernatant was filtered using a 0.22-μm membrane and was then injected into the UPLC system. Pooled quality control (QC) samples were prepared by mixing equal amounts (10 μL) of the supernatant after centrifugation and were run every 10 injections to evaluate the system stability and performance.

### UPLC–HRMS analysis

An ultrahigh performance liquid chromatography system (Thermo Fisher Scientific, Waltham, MA, USA) coupled online “via” a heated electrospray ionization source to a mass spectrometer was employed for nontargeted metabolomics profiling. Separation was achieved using an Acquity HSS T3 column (1.8 μm, 2.1 × 10 mm; Waters, Milford, MA, USA) with a 5-μL injection volume. The temperature of the chromatographic column was maintained at 40°C and that of the sample manager was maintained at 4°C. Gradient elution was performed using 0.1% formic acid in water (A) and acetonitrile (B) as the mobile phase. The flow rate was 0.3 mL/min, with the elution gradient as follows: 0–8 min, 2% B; 8–13 min, 50% B; 13–15 min, 85% B; 15–17 min, 98% B; 17–17.5 min, 2% B; and re-equilibration until 20.5 min. The acquisition of mass spectra was performed using a Q Exactive Orbitrap high-resolution mass spectrometer (Thermo Fisher Scientific) operated in positive and negative electrospray ionization modes with spray voltages of 3.5 and 3.0 kV, respectively. The capillary temperature was set at 350°C and the probe heating temperature was set at 300°C with the sheath gas set at 30 arbitrary units. The mass scan range was from 80 to 1 200 Da. The scanning mode was full scan/dd-MS^2^, and the mass resolution parameter for the full scan mode was 35 000 and 17 500 full width at half maximum (FWHM) for tandem mass spectrometry (MS/MS). The parameters of normalized collision energy were set to 12.5, 25, and 37.5 eV.

### Data processing and metabolite identification

Raw data (.raw) generated from the UPLC-HRMS analysis were initially processed using Compound Discoverer 3.0 (Thermo Fisher Scientific), including peak integration, nonlinear retention time alignment, filtering, and matching. Metabolite identification was based on accurately matching mass and mass fragmentation pattern spectra against the MS/MS spectra of metabolites available on the online databases, including mzCloud database (https://www.mzcloud.org); ChemSpider Web services (https://www.chemspider.com) and the Human Metabolome Database (HMDB, https://hmdb.ca/). The generated data matrix included information on the metabolite name, retention time, exact mass-to-charge ratio, and peak area. All data were imported into Excel (Microsoft, Redmond, WA, USA) for normalization of the peak areas.

### Metabolic profiling analysis

Pattern recognition was performed using SIMCA-P software (version 14.1; Umetrics AB, Umea, Sweden) to compare differences in the metabolic profile between the control and contused muscle groups. Principal component analysis (PCA) was used to observe outliers and the stability of the analytical system, and orthogonal partial least-squares discriminant analysis (OPLS-DA) was applied to discriminate the predicted wound age groups, similar to our previous study [4]. The potential biomarkers for differentiating the control group from the different wound age groups were selected based on their variable importance in projection (VIP) values from OPLS-DA and false discovery rate (FDR)-adjusted *P*-value. Heatmap and cluster analyses for the metabolites were performed using TBtool software (https://www.researchgate.net/deref/https%3A%2F%2Fgithub.com%2FCJ-Chen%2FTBtools%2Freleases). The metabolites significantly different in injured skeletal muscle were analysed using the pathway topology search tool in MetaboAnalyst 5.0 (https://www.researchgate.net/deref/https%3A%2F%2Fwww.metaboanalyst.ca).

### Machine-learning model development and evaluation

Before machine-learning model training, the *Z*-score standardization algorithm was used to preprocess the dataset to eliminate errors that originated from different dimensions between variables. The partial least-squares-based dimension reduction (PLS-DR) algorithm was applied to reduce the multidimensional dataset into a 2D dataset, which facilitates data visualization. This data conversion can overcome the high-dimension attribute of the metabolomics dataset and can extract as much helpful information from the data as possible.

Machine-learning algorithms were applied to learn data-distribution features from known data and predict anonymous data. Four machine-learning algorithms, logistic regression (LR), support vector machine (SVM), random forest (RF), and multilayer perceptron (MLP), were used in the present study. These machine-learning algorithms were mainly run on the Python 3.7 open-source platform with the scikit-learn module. In addition, the critical parameters of the four models were set as follows. Briefly, the LR solver was set as “lbfgs”, and the C-value that controls the regularization of the SVM model was set as 1.5 to improve fitting ability. Similarly, the n_estimators parameter of the RF model was set as 128, and the hidden_layer_sizes, solver, learning_rate, and max_iter parameters of the MLP model were set as (32, 32), “adam”, “adaptive”, and 3 000, respectively.

A crossvalidation algorithm was used to evaluate the performance of the four mathematic models in which the dataset was randomly divided into 70% training data and 30% validation data. Accuracy metrics were used to evaluate the performance of these models.

A tandem machine-learning model based on metabolomics data was established to predict wound age. First, contused muscle samples of 12 different ages (4, 8, 12, 16, 20, 24, 28, 32, 36, 40, 44, and 48 h) were divided into several groups based on OPLS-DA pattern recognition, and differential metabolites in the contused muscle were found based on their VIP value and adjusted *P*-value. Then, the metabolites from contused muscles were analysed to establish machine-learning models for predicting the wound age period. The best model with the highest performance among the four machine-learning algorithms was selected as the first-level prediction model. Second, the first-level groups were subdivided based on the OPLS-DA model outcomes, and the second-level prediction models were constructed based on their differential metabolites. Finally, we wrote code using Python software to link the two-level prediction models into a tandem model, which would be used for the prediction of new contused samples.

Another 13 rats were used to evaluate the tandem model performance for real-life application, and the extraction of contused muscle specimens and metabolomics analysis were performed as described above.

Finally, the accuracies of internal validation and Fisher discriminant analysis were used to evaluate the performance of the tandem model, which was compared with the performance of single machine learning models (LR, SVM, RF, and MLP, respectively).

## Results

### Metabolomics analysis and biomarker candidates in contused skeletal muscle

**Figure 1:**
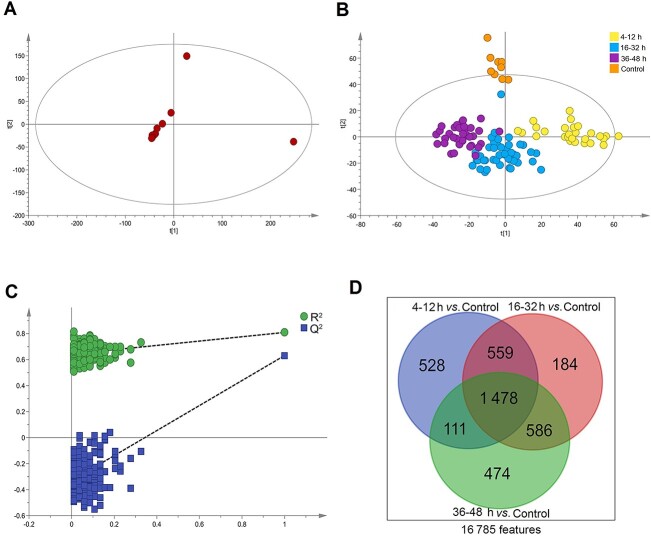
First-level classification of time intervals based on orthogonal partial least-squares discriminant analysis (OPLS-DA) pattern recognition and significant feature screening; (A) Principal component analysis (PCA) plot for quality control (QC) samples; (B) OPLS-DA pattern recognition for the control group and contused muscle groups; (C) permutation test for the OPLS-DA model to investigate whether this model was overfitted; (D) among 16 785 features, 3 920 significant features for the three contusion groups were identified.

A total of 16 785 chromatographic peaks (features) were obtained from UPLC–HRMS metabolomics data, which were applied for the multivariate analysis. The PCA score plot showed a close clustering of QC samples, indicating that satisfactory stability and reproducibility throughout the experiment protocol (Figure 1A). In addition, the OPLS-DA model in Figure 1B shows a complete separation of control group and contused skeletal muscle groups. Meanwhile, all contused skeletal samples from 12 of time intervals in the OPLS-DA model could be classified as three groups: Group I (4–12 h), Group II (16–32 h), and Group III (36–48 h). The model parameters (*R*^2^*Y* = 0.809, *Q*^2^ = 0.427, *P*-value of CV ANOVA < 0.001) and model validation (200 permutations, Figure 1C) demonstrated a good capacity for fitting and prediction.

To identify biomarkers associated with injury, the features with VIP > 1 in the OPLS-DA model and FDR-adjusted *P* < 0.05 were considered to be promising candidates for biomarkers. Compared with controls, there were 2 676 differentially expressed features in Group I (4–12 h), 2 807 differentially expressed features in Group II (16–32 h), and 2 649 differentially expressed features in Group III (36–48 h) (Figure 1D). As shown in Figure 1D, a total of 3 920 differential features in the three contused muscle groups were attributed to a panel of 43 endogenous metabolites (28 in Group I, 32 in Group II, and 28 in Group III) based on their accurately derived mass values, MS/MS spectra, and the parameters illustrated in Table 1.

**Table 1:** Detailed information of 43 differential metabolites found in contused muscle.

No.	*m*/*z*	Mass error (ppm)	Name	Formula	Retention time (min)	mzCloud ID	HMDB ID
1	327.3135	−0.9	*N*,*N*-dimethylsphingosine	C_20_H_41_NO_2_	22.302	4 434	HMDB0013645
2	453.2856	0.2	Glycerophospho-*n*-palmitoyl ethanolamine	C_21_H_44_NO_7_P	16.403	2 955	NA
3	204.0899	0.2	DL-tryptophan	C_11_H_12_N_2_O_2_	8.241	415	HMDB0013609
4	117.0789	−0.5	L-(+)-valine	C_5_H_11_NO_2_	0.880	2 570	HMDB0000883
5	187.0633	0.0	Indoleacrylic acid	C_11_H_9_NO_2_	8.240	449	HMDB0000734
6	125.0146	−0.2	Taurine	C_2_H_7_NO_3_S	0.852	654	HMDB0000251
7	344.2355	1.0	Medroxyprogesterone	C_22_H_32_O_3_	17.385	1 321	HMDB0001939
8	164.0474	0.2	4-Coumaric acid	C_9_H_8_O_3_	1.086	539	HMDB0002035
9	181.0739	0.2	L-tyrosine	C_9_H_11_NO_3_	1.078	2 255	HMDB0000158
10	320.2354	0.9	18-HETE	C_20_H_32_O_3_	17.521	4 720	HMDB0006245
11	408.2882	1.5	Cholic acid	C_24_H_4_0O_5_	13.096	344	HMDB0000619
12	149.0511	0.1	L-(−)-methionine	C_5_H_11_NO_2_S	1.080	1 544	HMDB0000696
13	296.2354	0.8	13S-hydroxyoctadecadienoic acid	C_18_H_32_O_3_	16.744	9 778	HMDB0004667
14	244.0698	1.0	Uridine	C_9_H_12_N_2_O_6_	1.853	1 408	HMDB0000296
15	278.2244	−0.7	α-Eleostearic acid	C_18_H_30_O_2_	16.753	6 594	HMDB0248208
16	168.0283	−0.1	Uric acid	C_5_H_4_N_4_O_3_	1.076	753	HMDB0000289
17	321.2666	−0.7	α-Linolenoyl ethanolamide	C_20_H_35_NO_2_	17.587	4 417	HMDB0013624
18	378.2768	−0.6	2-Arachidonoylglycerol	C_23_H_38_O_4_	20.076	4 740	HMDB0004666
19	131.0947	0.4	Leucine	C_6_H_13_NO_2_	0.357	6	HMDB0000687
20	392.2933	1.5	3a,7a-dihydroxycholanoic acid	C_24_H_40_O_4_	15.084	9 830	HMDB0000384
21	449.3145	0.9	Glycochenodeoxycholic acid	C_26_H_43_NO_5_	13.513	9 839	HMDB0000637
22	272.2356	1.5	Juniperic acid	C_16_H_32_O_3_	20.262	2 551	HMDB0006294
23	320.2354	0.9	15-HETE	C_20_H_32_O_3_	17.069	8 581	HMDB0003876
24	112.0272	−0.6	Uracil	C_4_H_4_N_2_O_2_	1.845	2 531	HMDB0000300
25	392.2933	−0.9	Deoxycholic acid	C_24_H_40_O_4_	15.400	388	HMDB0000626
26	230.1518	0.1	Dodecanedioic acid	C_12_H_22_O_4_	11.170	1 182	HMDB0000623
27	584.2630	−0.8	Bilirubin	C_33_H_36_N_4_O_6_	25.613	334	HMDB0000054
28	302.2244	−0.5	Eicosapentaenoic acid	C_20_H_30_O_2_	20.345	348	HMDB0001999
29	165.0790	0.1	DL-phenylalanine	C_9_H_11_NO_2_	1.153	8	HMDB0250791
30	166.0630	0.0	Desaminotyrosine	C_9_H_10_O_3_	9.922	183	HMDB0002199
31	312.2299	−0.6	9-HpODE	C_18_H_32_O_4_	12.722	8 563	HMDB0242602
32	354.2412	1.6	(−)-Prostaglandin E1	C_20_H_34_O_5_	13.359	1 356	HMDB0001442
33	304.2400	−0.8	Arachidonic acid	C_20_H_32_O_2_	17.734	2 742	HMDB0001043
34	286.2148	1.2	Hexadecanedioic acid	C_16_H_30_O_4_	13.304	2 712	HMDB0000672
35	185.1052	0.1	Ecgonine	C_9_H_15_NO_3_	9.746	2 066	HMDB0006548
36	328.2254	1.4	(11E,15Z)-9,10,13-trihydroxyoctadeca-11,15-dienoic acid	C_18_H_32_O_5_	11.572	1 416	NA
37	298.2506	−0.8	NP-011548	C_18_H_34_O_3_	19.620	6 496	NA
38	238.2295	−0.6	Muscone	C_16_H_30_O	22.893	6 153	HMDB0034181
39	299.2821	−1.1	D-sphingosine	C_18_H_37_NO_2_	15.563	418	HMDB0000252
40	159.0896	0.2	*N*-acetylvaline	C_7_H_13_NO_3_	9.480	1 365	HMDB0011757
41	301.2979	−0.6	Sphinganine	C_18_H_39_NO_2_	11.921	391	HMDB0000269
42	281.2718	−0.4	(9Z)-9-octadecenamide	C_18_H_35_NO	24.440	530	HMDB0002117
43	176.0321	0.2	Vitamin C	C_6_H_8_O_6_	11.161	325	HMDB0000044

NA: not available.

**Figure 2:**
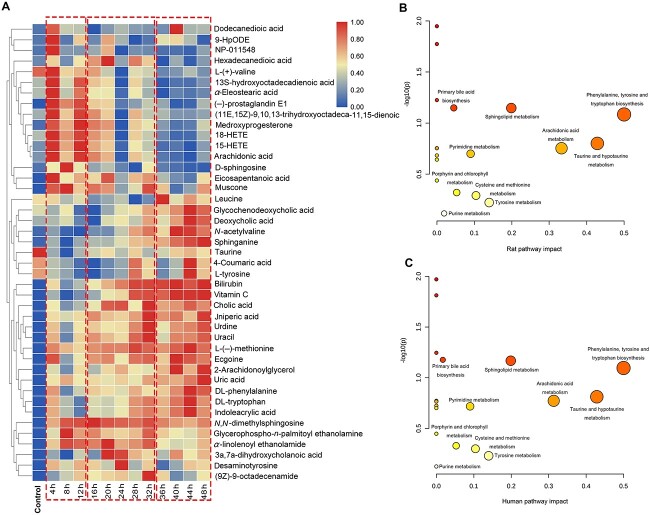
Hierarchical cluster analysis and pathway analysis; (A) hierarchical cluster plot of a panel of 43 metabolites identified in the wound age groups; view maps of the metabolic pathways in rat (B) and human (C); this figure displays all matched pathways as circles; the *x*-axis represents enriched pathways, and the *y*-axis represents the impact pathways; the colour and size of each circle are based on its *P*-value and the pathway impact value, respectively.

Furthermore, hierarchical cluster plots of the 43 metabolites were constructed (Figure 2A), in which blue indicates a low level and red indicates a high level, showing changes in the levels of metabolites in skeletal muscle after injury. Significant differences were observed between the three wound age periods (4–12 h, 16–32 h, and 36–48 h). Forty-three differential metabolites were identified in the skeletal muscle of injured rats, 40 of which were common to rat and human (Table 1), based on HMDB comparison. Pathway analyses for these 40 metabolites showed that 10 perturbed common metabolic pathways in both species were obtained from the pathway libraries of *Rattus norvegicus* (rat) and *Homo sapiens* (human) using MetPA (Figure 2B and C), and they were correlated with the metabolisms of lipid, amino acid, and nucleotide.

### Establishment of the first-level prediction model to distinguish wounds of different ages

**Figure 3:**
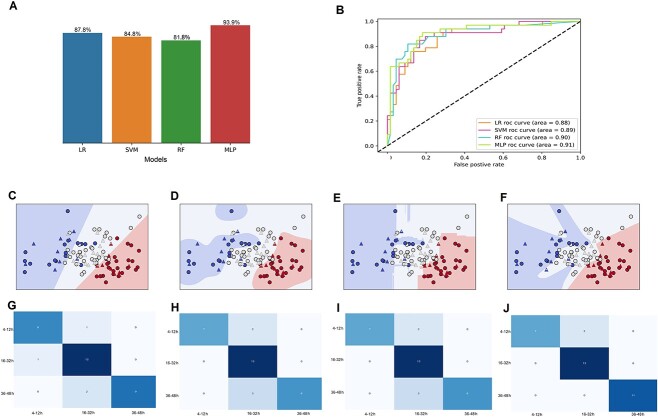
The assessment of the first-level prediction model; (A) the validation accuracies of the crossvalidation in logistic regression (LR), support vector machine (SVM), random forest (RF), and multilayer perceptron (MLP); (B) receiver operator characteristic (ROC) curves and area under the curve values of the four algorithms; classification hyperplane plots of the LR (C), SVM (D), RF (E), and MLP (F) algorithms; the blue area represents the data space of the 4–12-h group, the white area represents the 16–32-h group, and the red area represents the 36–48-h group; round dots represent training samples, triangles represent validation samples, and pentagrams represent misjudged samples; (G–J) confusion matrix of 4–12-, 16–32-, and 36–48-h groups in LR, SVM, RF, and MLP algorithms, respectively.

The first-level prediction model was constructed based on the preliminary separation of time intervals using OPLS-DA. Firstly, the PLS-DR algorithm was introduced to reduce the dataset dimensions, and the 43D dataset was projected onto two dimensions. Then, four prediction models based on LR, SVM, RF, and MLP algorithms were established to discriminate the samples obtained at 4–12 h (Group I), 16–32 h (Group II), and 36–48 h (Group III). The validation accuracies of the crossvalidation were 87.8%, 84.8%, 81.8%, and 93.9% (Figure 3A) and the area under the curve values of the receiver operating characteristic (ROC) curve were 0.88, 0.89, 0.90, and 0.91 for LR, SVM, RF, and MLP algorithms, respectively (Figure 3B). The classification hyperplane and confusion matrix plots are shown in Figure 3C–J, indicating the separation of samples and the number of correctly judged and misjudged samples in each model. According to the results, the MLP model achieved a higher performance compared with the LR, SVM, and RF models, and it was selected as the first-level prediction model to discriminate wounds of different ages.

### Establishment of the second-level prediction model for estimating wound age time interval

**Figure 4:**
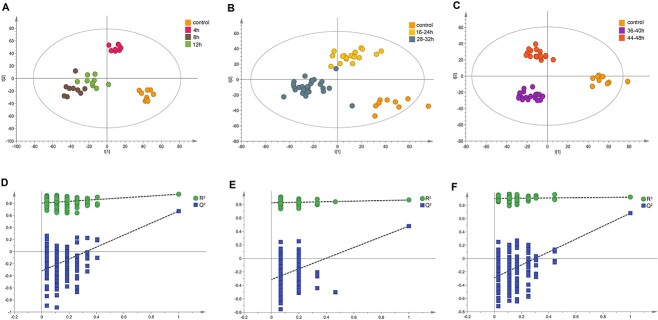
Second-level division of time intervals based on orthogonal partial least-squares discriminant analysis (OPLS-DA) pattern recognition; OPLS-DA plots indicating that discrimination between 4-, 8-, and 12-h samples (A); 16–20- and 24–32-h samples (B); and 36–40- and 44–48-h samples (C) was possible; (D–F) permutation test results (200 times) for each model.

On the basis of the wound age, OPLS-DA was performed to further subdivide the contused skeletal muscle samples within above three groups. As illustrated in scatter plots (Figure 4A–C), the muscle samples in Group I were divided into three subgroups—4, 8, and 12 h; Group II samples were divided into 16–20-h and 24–32-h subgroups; and Group III samples were divided into 36–40-h and 44–48-h subgroups. The parameters for these models (*R*^2^*Y* = 0.907, 0.852, and 0.945, *Q*^2^ = 0.458, 0.470, and 0.455, and *P*_CV-ANOVA_ = 0.00215979, 5.41912e^−008^, and 2.5132e^−005^, respectively) and permutation test results (200 permutations, Figure 4D–F) indicated that the OPLS-DA models performed well and were not overfitted.

**Figure 5:**
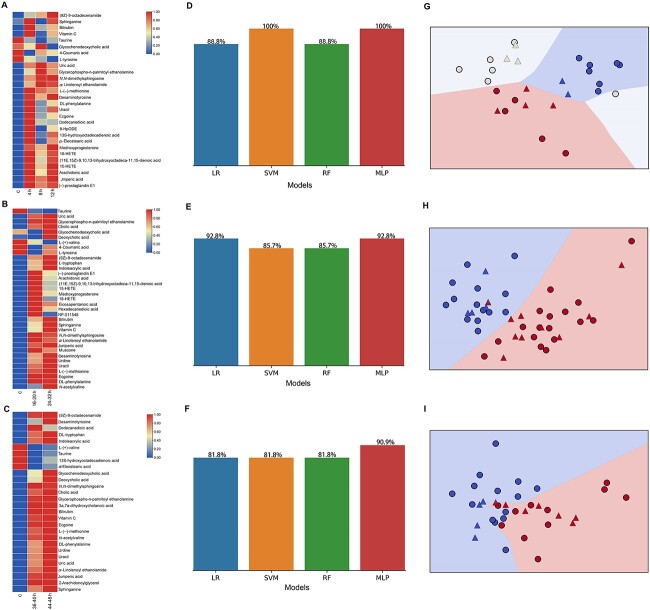
The assessment of the second-level prediction model; heatmaps representing average metabolite levels in 4-, 8-, and 12-h groups (A); 16–20- and 24–32-h groups (B); and 40–44- and 44–48-h groups (C); the crossvalidation accuracies of the four models in 4-, 8-, and 12-h groups (D); 16–20- and 24–32-h groups (E); and 40–44- and 44–48-h groups (F); MLP classification hyperplane plots for second-level temporal division for 4-, 8-, and 12-h groups (G) (blue, red, white areas); 16–20- and 24–32-h groups (H) (blue and red areas); and 40–44- and 44–48-h groups (I) (blue and red areas); round dots represent training samples; triangles represent validation samples.

Based on these OPLS-DA models, differential metabolites with VIP>1 and adjusted *P*-value<0.05 were screened again. Ultimately, compared to the control group, 28 differential metabolites were found in 4-, 8-, and 12-h groups, 35 metabolites were found in 16–20- and 24–32-h groups, and 28 metabolites were found in 36–40- and 44–48-h groups. These metabolites were the consistent in the above-listed 43 metabolites identified by the first-level model (Table 1). The heatmaps plotted in Figure 5A–C showed distinct differences among the subgroups.

Four models, LR, SVM, RF, and MLP, were established to predict second-level divisions of time intervals based on their respective metabolite panel. Similar to the above method, the PLS-DR algorithm was also applied for dimensional reduction to project the dataset onto two dimensions before model training. According to its accuracy level (Figure 5D–F), the MLP algorithm exhibited the highest discriminatory efficiency for second-level temporal division among the four models after crossvalidation. The hyperplane plots of the MLP model revealed consistent accuracy and classification results (Figure 5G–I). The contused muscle samples can be divided into seven subgroups (4, 8, 12, 16–20, 24–32, 36–40, and 44–48 h) by the second-level tandem model, indicating that the time windows for wound age have a minimum interval of 4 h and a maximum interval of 12 h.

### Establishment and validation of the tandem prediction models

**Figure 6:**
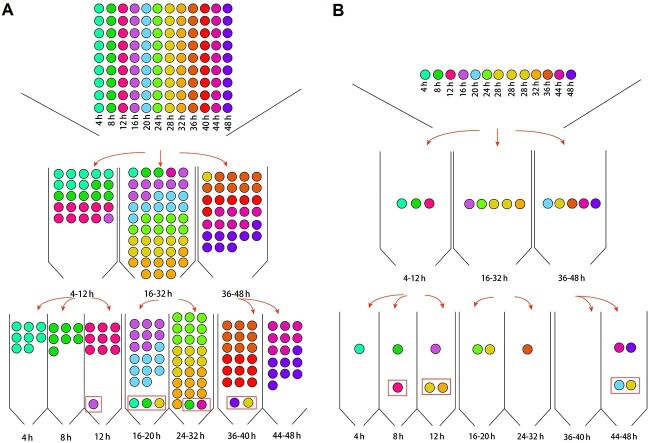
Workflow of internal validation (A) and external validation (B) of the MLP–MLP tandem prediction model; samples from different groups are denoted with distinct colours, and points within red boxes represent misjudged samples.

Based on the above results, the two-level MLP classification model consisting of four MLP models was efficient at estimating the wound ages of contused skeletal muscle samples obtained from rats. For more convenient real-life application, detailed Python code was written to concatenate these first-level MLP model and second-level MLP models into a tandem prediction model, with uploaded code in GitHub repository (https://github.com/asdwe172009/tandem-machine-learning-model-for-rats-skeletal-muscle-wound-age-estimation). In this section, data from the 108 samples (12 contused time point, *n* = 9) in the experiment were input into a novel MLP–MLP tandem model to validate the internal performance of the integrated tandem model. Consequently, 100 of 108 samples were correctly discriminated (Figure 6A), demonstrating the robust performance of the tandem model.

To evaluate tandem model performance using the test dataset, samples from another 13 rats collected at different times after injury were designated out-of-bag data for a double-blind experiment. The metabolomics experiment and data preprocessing were entirely consistent with those used for the training samples. Then, the test data were input into the tandem machine-learning model for classification. The workflow of the classification process is shown in Figure 6B, which indicated the analysis workflow of unknown samples in the tandem model. In this experiment of external validation, eight of the 13 samples were correctly discriminated.

**Figure 7:**
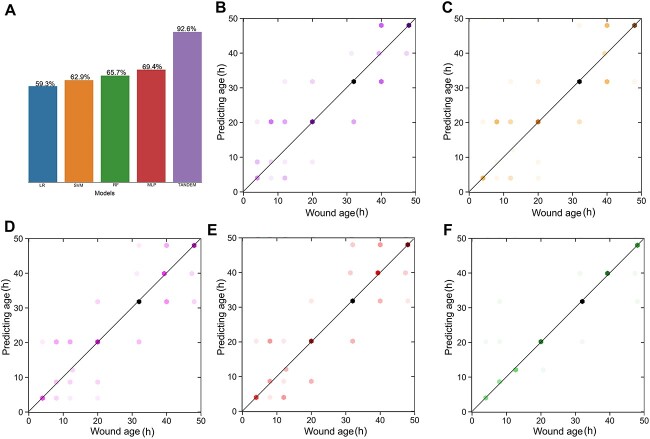
The results of comparison between models; (A) the accuracies of internal validation for logistic regression (LR), support vector machine (SVM), random forest (RF), multilayer perceptron (MLP), and MLP–MLP tandem model; the scatter plots of predicting age and actual wound age in each model; (B) LR; (C) SVM; (D) RF; (E) MLP; (F) MLP–MLP tandem model; one dot represents one or more sample; the more points that fall in the same position, the darker the colour; the points on the line are the samples with the correct prediction.

To compare with the performance of single model and tandem model, LR, SVM, RF, and MLP were used for modelling, respectively. As shown in Figure 7A, the tandem model achieved an accuracy of 92.6%, which was much higher than all accuracy of the four single machine learning models (59.3% for LR, 62.9% for SVM, 65.7% for RF, and 69.4% for MLP). The scatter plot of predicting age and actual wound age showed the prediction ability of each model (Figure 7B–F). In Figure 7F, the most numerous points gathered diagonally, which indicated that the MLP–MLP tandem model was more suitable for wound age estimation than single models.

## Discussion

Estimating the amount of time elapsed since injury has been a goal in traditional forensic medicine over the past decades. After extensive research [4, 9, 10], forensic scholars have agreed that multiple biomarkers should be used to estimate the time since injury [22]. This agreement has received much attention because the reduction of errors is important. However, the identification of good biomarkers for the screening and analysis of relevant data for inferring time elapsed from injury remain challenging. Metabolomics, the high-throughput analysis of small molecules in biological materials, can reveal biologically relevant perturbations in metabolite profiles that result from diseases [23–25], and the filtering of biomarkers at the metabolic level is inherent for this type of analysis [26]. However, the metabolites reflecting various wound ages remain unknown. In the present study, metabolic biomarkers significantly different from the control group were first screened, and a panel of 43 metabolites found in contused skeletal muscle was identified.

Changes after skeletal muscle injury involve the progress of inflammation, namely, increases in vascular permeability, neutrophil exudation, and tissue regeneration and repair [8]. When reviewing the literature, we found that several of the 43 metabolites identified in this article are involved in the injury repair process. For example, prostaglandin E1 has been reported to regulate blood flow, vascular permeability, and neutrophil migration as a chemokine [27], and 13S-hydroxyoctadecadienoic acid regulates intercellular adhesion as inflammation progresses [28]. Notably, the changes in prostaglandin E1, 15-HETE, 18-HETE, 13S-hydroxyoctadecadienoic acid, and medroxyprogesterone exhibited similar trends, which is in agreement with the research of Markworth et al. [27]. The above results indicated that the metabolites identified in the present study are highly correlated with skeletal muscle injury and can be applied for the wound age estimation.

In several preliminary studies of wound age estimation, multibiomarker analysis was suggested to improve wound age estimation sensitivity and specificity [1, 22, 29, 30]. In the previous studies, multibiomarker analysis was used at morphological level according to the presence or absence of biomarkers [9, 10], or at the genetic level according to several biomarkers level changes, such as “up, no change, and down”, to estimate the wound age [22, 30]. However, the above data analysis approach forcibly transforms continuous variables into discrete variables, resulting in the loss of valuable information from biomarkers. According to the results of present study, machine learning models, which may consider the relationship between different biomarkers, provided a more efficient method for wound age estimation.

Metabolomics is a high-throughput technology and inevitably produces a large number of high-dimensional datasets, which may result in serious overfitting when machine-learning models are established [31, 32]. In a previous study, PCA, as an unsupervised dimension reduction algorithm, was considered in machine-learning modelling [32]. However, this dimension reduction method is not capable of handling high-dimensional datasets derived from multiple time points. Thus, a powerful dimension reduction method, the PLS-DR algorithm, was employed in this experiment because a supervised algorithm always achieves better performance than an unsupervised algorithm [33].

In the present research, the time window for wound age was narrowed to a minimum of 4 h, which surpassed the result obtained in our previous study [4]. Firstly, merging groups and regrouping wound age groups is an effective method to improve model performance. Moreover, this result is also due to the application of MLP–MLP tandem machine-learning models in the present study. As seen from the results in Figures 3 and 5, although LR, SVM, and RF models were also established, the MLP algorithm exhibited a relatively robust performance for all classification tasks. This outcome may be because the MLP model is a deep-learning model, which always gives a better and more robust performance compared to shallow models [34, 35].

Classification and regression models are the two main approaches for the inference of temporal events in forensics [36–40]. Regression models are used for estimating time since death in the previous studies [17, 41], with the result expressed as a time point plus or minus the root-mean-square error or standard deviation. Simultaneously, regression models also group postmortem timepoints into many periods [42], similar to classification models for practical application. In the present study, the wound ages of samples were classified into 4-, 8-, 12-, 16–20-, 24–32-, 36–40-, and 44–48-h groups based on the pattern recognition results from OPLS-DA, which may produce results with greater biological significance compared to regression models based only on the learning performance from training samples.

As we all know, to circumvent limitations associated with human studies (mostly limited to ethic reasons and heterogeneity between individuals), animal model studies could be conducted first. In the present study, whether the result from rats could be applied to human depends on whether there are similar metabolic response patterns in the two species. Park et al. [43] demonstrated that a lot of common endogenous metabolites were present between primates and rodents, and many metabolites with low interspecies variability could be identified for bioeffect monitoring. In the study of deep venous thrombosis, we also found that humans and rats share a great number of common metabolites and relative metabolic pathways associated with disease [44]. These studies indicated that metabolites within the same class are unlikely to be species-specific, which would be useful to translate the findings in rodent models to human [45, 46].

In the study of sudden cardiac death, the MLP model, which based on rat metabolomics dataset, achieved an accuracy of 88.23% and a ROC of 0.89 for predicting the acute myocardial ischemia (AMI) type II in autopsy cases of sudden cardiac death, verifying the feasibility of cross-species metabolomics [47]. In the present study, there were 40 metabolites and 10 metabolic pathways identified in the injured skeletal muscle of rat after injury, which were common to human. Therefore, although there is much difference in biological phenomena between rats and humans, there would be similar expression patterns of the endogenous metabolites in the two species. Cross-species metabolomics can generate associations among specific metabolites, which would help to translate the current results from rats to human applications by probing correction relationships based on these metabolites.

Although combining metabolomics approach and machine-learning algorithm to establish prediction models has been demonstrated as a strategy potentially useful for wound age estimation in present study, the model will be more accurate and reliable if a larger sample size was used, and the postmortem intervals, temperatures, and humidity would be performed to refine the prediction models in future research.

## Conclusion

A panel of 43 differential metabolites found in contused skeletal muscle was identified using metabolomic analysis in the present study. Then, an MLP–MLP tandem machine-learning model was established based on these metabolites to determine wound age intervals. The model achieved a prediction accuracy of 92.6%, which was much higher than that of the single model. The time window for wound age estimation had a minimum interval of 4 h and a maximum interval of 12 h. Results presented in this report revealed that combination of metabolomics approach and machine-learning algorithms could be a novel strategy for wound age estimation in future forensic casework.

## Authors' contributions

Jie Cao, Guoshuai An and Junhong Sun conceived the idea and drafted the manuscript. Jian Li and Liangliang Wang carried out the metabolomics experiment and performed the statistical analysis. Kang Ren established animal model. Qiuxiang Du performed data collection. Keming Yun and Yingyuan Wang reviewed the manuscript. All authors have read and agreed to the published version of the manuscript.

## Compliance with ethical standards

This study was approved by the Animal Ethics Committee of Shanxi Medical University, China (2016LL151). All procedures performed conformed to the Guide for the Care and Use of Laboratory Animals (https://nap.nationalacademies.org/catalog/12910/guide-for-the-care-and-use-of-laboratory-animals-eighth) and were approved by the Institutional Animal Care and Use Committee of Shanxi Medical University, China.

## Data availability

The datasets generated during and/or analysed during the current study are available from the corresponding author on reasonable request. The Python code for establishment of machine-learning model and corresponding datasets were uploaded to the Github repository: https://github.com/asdwe172009/tandem-machine-learning-model-for-rats-skeletal-muscle-wound-age-estimation.

## Disclosure statement

The authors report there are no competing interests to declare.

## Funding

This work was supported by the National Natural Science Foundation of China [number 81901924 and 81971795].
